# The TIGER/Islander resource for genomic islands

**DOI:** 10.1128/mra.00492-25

**Published:** 2025-08-11

**Authors:** Steven L. Yu, Hannah M. McClain, Ellis L. Torrance, Joseph S. Schoeniger, Catherine M. Mageeney, Kelly P. Williams

**Affiliations:** 1 Sandia National Laboratories 1105, Livermore, California, USA; University of California Riverside, Riverside, California, USA

**Keywords:** genomic island, sequence database, comparative genomics, bioinformatics

## Abstract

Genomic islands (GIs) are integrative mobile DNAs shared among bacteria or archaea, often bringing cargo genes that affect bacterial phenotypes like virulence and metabolism; many are prophages with utility in therapy. We applied our TIGER and Islander software to ~460,000 prokaryotic genomes, yielding a large database containing ~1.75 million GIs.

## ANNOUNCEMENT

**Fig 1:**
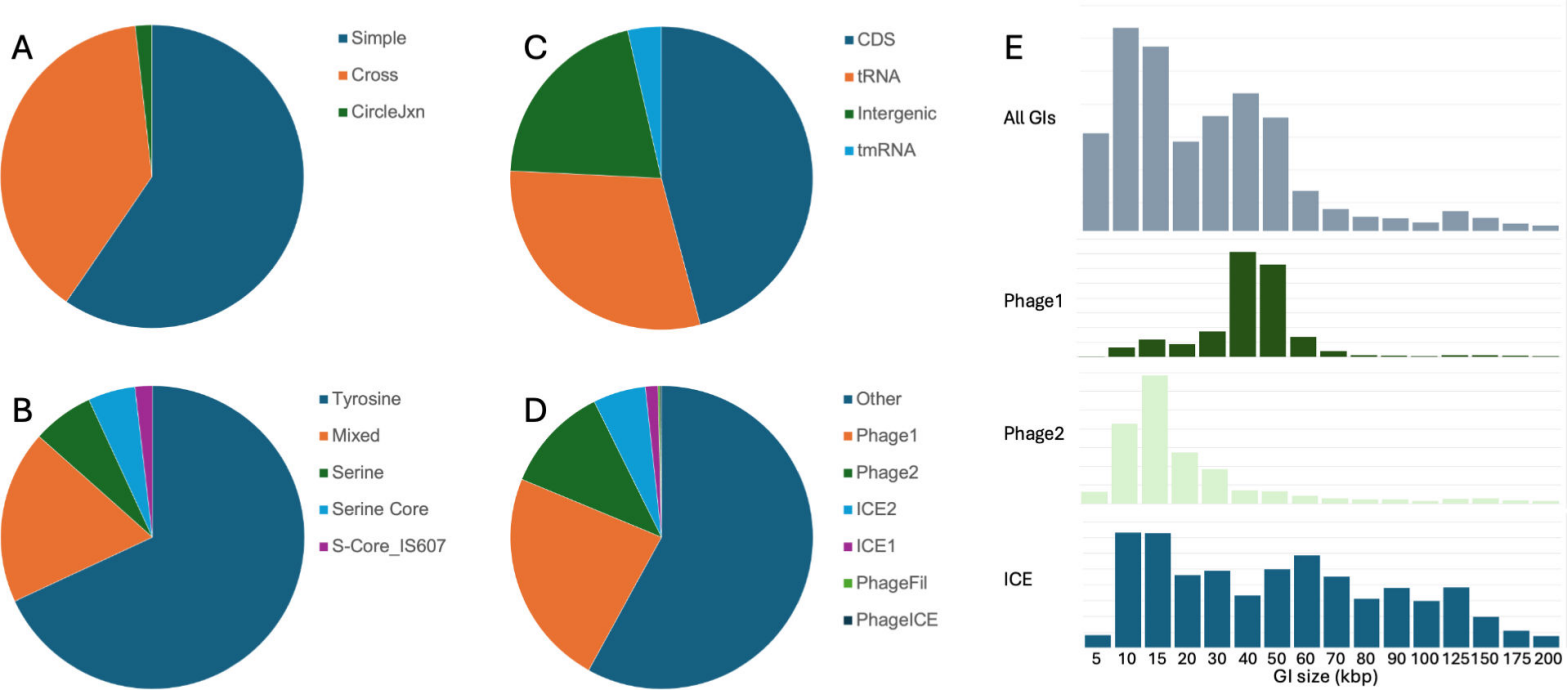
Statistics of GI assignments. (A). Composition of the GI. TIGER allows detection of split (cross-contig or circle-junction) GIs or simple GIs internal to a single contig. (B). Integrase type used in each genomic island. “Serine core” is typically non-integrase lacking the characteristic recombinase domain and is usually known as invertase or resolvase. “S-Core_IS607” is a clade with site-promiscuity associated with the insertion sequence IS607. (C). Integration site. CDS: protein-coding sequence. (D). GI type based on gene content. (E). GI length distribution. The top panel is for the entire data set and lower panels are for the Phage1, Phage2, and combined ICE1/2 types.

Genomic Islands (GIs), a diverse class of mobile genetic elements encoding integrases, promote horizontal gene transfer in bacteria and archaea. Identification and precise mapping of GIs has been challenging: their gene content is often poorly understood, and it is difficult to identify and validate their genomic endpoints. However, precise mapping provides insights into mechanisms of transfer and integration, identifies prophages useful for various applications (1), reveals novel cases of regulated gene integrity (2), and enables discovery and characterization of auxiliary metabolic genes, virulence factors, and other genes that alter host phenotype (3). Improved knowledge of GI sequences and genes also facilitates characterization of host–GI defense systems (4) and development of novel tools for synthetic biology (e.g., integrases [5], CRISPR systems [6], polymerases [7, 8]). We aimed to develop the most precise and phylogenetically comprehensive prokaryotic GI database possible with current data. We downloaded 458,681 prokaryotic genomes from GenBank that could be assigned to species in Genome Taxonomy Database (GTDB) release 214, as described in reference (9). We applied two orthogonal GI-finding algorithms notable for their precision in mapping GI termini: Islander 1.0 (10), limited to finding single-contig islands in tRNA/tmRNA genes, and TIGER 2.0 (2, 11, 12), which compares to reference genomes and can detect cross-contig islands. The latter program was run in “circle-junction” mode for presumably complete genomes with five or fewer contigs, or otherwise in “cross-contig” mode. Cross-contig islands have precisely defined termini but may be missing internal sequences. Additional software in TIGER 2.0 resolved overlaps and tandem arrays. GIs <2 kbp and >200 kbp were excluded. This process yielded 1,757,053 island assignments, likely including some false positives. The majority of GIs have simple (internal to a single contig) structure (Fig. 1A). Our software insists that GIs contain at least one integrase gene, most commonly in the tyrosine recombinase family (68%, Fig. 1B). Among GIs containing integrases in the serine recombinase family, the Serine Core group (lacking the Pfam domain “Recombinase” characteristic of integrases) is usually annotated as DNA invertases or resolvases; Serine Core GIs may be false positives or reveal novel Recombinase-lacking integrases or use of exogenous helper integrases. The site-promiscuous IS607 subfamily is known to mobilize a group of transposon-like elements (13). Our precise detection methods specify the locus of integration for each GI. We find that 33.8% of GIs integrate into a tRNA or tmRNA gene, 43.4% are in protein coding sequences, and 22.8% are intergenic (Fig. 1C). Gene content categorized our GIs as 34.9% prophages and 7.0% Integrative and Conjugative Elements (ICEs), and <0.1% as phage-ICE tandems. The remaining 58% were not readily categorizable by current gene content algorithms (Fig. 1D). There are two prominent peaks in the GI size distribution, 10 kbp and 40 kbp; the latter is attributed to our top category of prophages (Phage1) (Fig. 1E). The database provides a large, diverse survey of prokaryotic GIs that is unique for the precision of integration site mapping.

## Data Availability

The database is available as a flat file at https://figshare.com/s/fe1d563b00782d187880 (file extension .tsv.gz; size 108.4 Mb, inflating to 635.4 Mb). Also at that website is a table listing the genomes analyzed.
